# The Cause of Ribbon Fluctuation in Magnetorheological Finishing and Its Influence on Surface Mid-Spatial Frequency Error

**DOI:** 10.3390/mi13050697

**Published:** 2022-04-29

**Authors:** Bo Wang, Feng Shi, Guipeng Tie, Wanli Zhang, Ci Song, Ye Tian, Yongxiang Shen

**Affiliations:** 1 College of Intelligence Science and Technology, National University of Defense Technology, 109 Deya Road, Changsha 410073, China; wbnudt@163.com (B.W.); shifeng@nudt.edu.cn (F.S.); zhangwanli17@nudt.edu.cn (W.Z.); songci@nudt.edu.cn (C.S.); tianyecomeon@sina.cn (Y.T.); xiangyueni@163.com (Y.S.); 2 Hunan Key Laboratory of Ultra-Precision Machining Technology, Changsha 410073, China; 3 Laboratory of Science and Technology on Integrated Logistics Support, National University of Defense Technology, 109 Deya Road, Changsha 410073, China

**Keywords:** high-power laser component, magnetorheological finishing, ribbon fluctuation, mid-spatial frequency error

## Abstract

In the high-power laser system, the mid-spatial frequency error of the surface of the high-power laser component will affect the normal operation of the high-power laser system. In order to improve the mid-spatial frequency error of the high-power laser component after magnetorheological finishing, the causes and influencing factors of the ribbon fluctuation in magnetorheological finishing are studied, and the influence of different ribbon fluctuation on the mid-spatial frequency error of the surface is studied. Firstly, the influence of different ribbon fluctuations on the mid-spatial frequency error of the machined surface is simulated by a computer. Secondly, the magnetic field in the circumferential direction of the polishing wheel, the fluctuation amount and frequency of the magnetorheological polishing ribbon are measured, and then the causes of the fluctuation of the magnetorheological polishing ribbon are analyzed. Moreover, through the principle of a single variable, the influence of process parameters on the fluctuation of magnetorheological polishing ribbon is explored. Finally, the fused silica component is scanned uniformly under the process parameters of magnetorheological polishing ribbon fluctuation of 40 μm, 80 μm, 150 μm, and 200 μm. The experimental results show that the greater the ribbon fluctuation, the greater the surface mid-spatial frequency error of the component, and the ribbon fluctuation is approximately linear with the RMS of the PSD2 in the mid-spatial frequency band on the surface of the component. Therefore, the fluctuation of the ribbon can be controlled by controlling the magnetorheological processing parameters, and the mid-spatial frequency band error on the surface of the high-power laser component can be significantly reduced by optimizing process parameters after magnetorheological finishing.

## 1. Introduction

The high-power laser device used in inertial confinement laser fusion has a huge demand for large aperture planar optical components. For example, Lawrence Livermore National Key Laboratory (LLNL) in the United States, its National Ignition Facility (NIF), includes 192 laser beams and more than 2000 planar optical components with an aperture greater than 400 mm [1]. In addition to the demand for these optical components in terms of quantity, there are also high requirements for the spectral error of these planar optical elements in the full frequency band, especially in the mid-spatial frequency band. LLNL divides the surface error of the large aperture plane element of the NIF system into three different frequency bands: low, middle, and high. Among them, the spatial frequency of the spectral error in the mid-spatial frequency band is 0.0303–8.333 mm^−1^, and the evaluation method based on power spectral density is adopted. The middle frequency band is subdivided into PSD1 and PSD2, and the spatial frequencies of the two bands are 0.0303–0.4 mm^−1^ and 0.4–8.333 mm^−1^, respectively. NIF requires that the RMS of the PSD1 band is less than 1.8 nm and that of the PSD2 band be less than 1.1 nm. The influence of the mid-spatial frequency error on the laser system is mainly reflected in the nonlinear self-focusing caused by the increase in the modulation system of the beam, which leads to the filament destruction of the components and endangers the safe operation of the laser system [2]. Most importantly, once the MSF error is generated, it is difficult to eliminate completely, so this error has become the main technical bottleneck that hinders the development of optical polishing [3].

Magnetorheological finishing (MRF) technology uses the flexible polishing tool formed by the rheological effect of magnetorheological fluid in the magnetic field to process the optical components [4]. At present, this technology has been widely used in polishing various optical materials; the advantage of this method is its high reliability, and there is no damage to the surface and sub-surface [5]. The magnetorheological fluid in MRF consists of magnetic carbonyl iron particles, non-magnetic polishing abrasives, and water or other non-carrier fluids and stabilizers [6]. Since the high-intensity gradient magnetic field is distributed in the gap between the polishing wheel and the workpiece, when the magnetorheological fluid flows through the polishing gap, it produces a rheological effect and then becomes a Bingham liquid and forms a polishing ribbon. Driven by the polishing wheel, the polishing ribbon will generate positive pressure and shear force on the workpiece surface and, finally, realize the material removal on the component surface [7,8]. However, the instability of the magnetorheological polishing ribbon leads to the instability of the transient removal function during processing, which will eventually form the mid-spatial frequency band error on the component surface.

**Figure 1:**
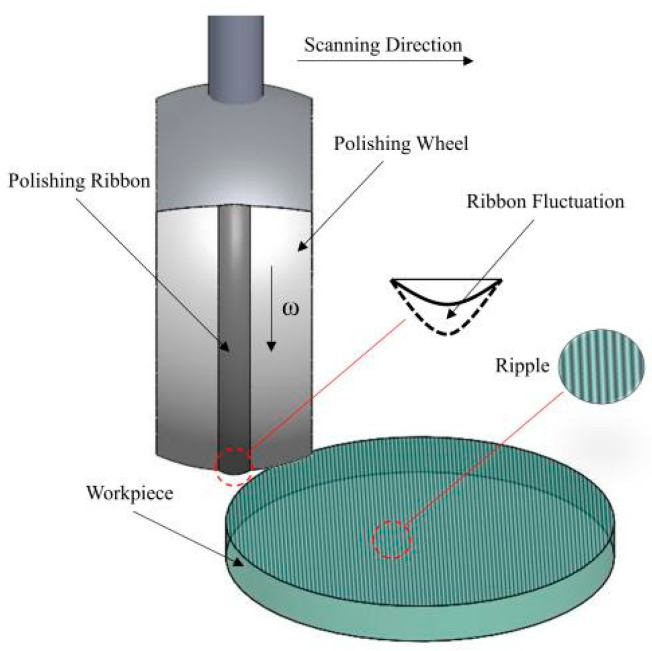
Schematic diagram of the mid-spatial frequency error caused by ribbon fluctuations on the component surface during magnetorheological finishing.

In the process of magnetorheological finishing, since the size of the polishing spot we used is much smaller than that of the machining element, many small-scale ripple errors, namely mid-spatial frequency errors, will be introduced when the low-frequency errors of the machined surface are quickly removed [9]. At present, the main factors affecting the residual error of the magnetorheological finishing process include: (1) The initial surface error: Error distribution of initial surface in the spatial domain. (2) Removal function: The size of the removal function, removal rate, and removal stability directly affect the residual error after machining. (3) Dwelling time: The dwelling time of each point directly affects the removal amount of the point. (4) Trajectory planning: Trajectory planning will affect the convolution error and thus affect the surface quality [10]. The research on the mid-spatial frequency error of magnetorheological finishing surfaces has made great progress in recent years. In order to suppress the periodic texture of the optical surface, Dunn and Walkerd developed a random path planning algorithm, which can improve the MSF error [11]. Hu et al. proposed a step adaptive Archimede path to reduce the mid-spatial frequency error on the component surface by adjusting the path step according to the material removal amount [12]. Wang et al. analyzed the effect of the tool step size on the TPR_error in sub-aperture deterministic polishing (SDP) and studied a method that can optimize the tool step size to restrain this error [13]. In terms of tool path, Wang et al. proposed a novel unicursal random maze tool path, which can not only implement uniform coverage of the polishing surfaces but also possesses randomness and multidirectionality [14]. Yang et al. proposed the random row spacing method to suppress the high-frequency error, theoretically analyzed the generation mechanism of the mid-spatial frequency and high-frequency error in the fixed row spacing method of magnetorheological finishing, and verified the effectiveness of the random row spacing method in suppressing the mid-spatial frequency and high-frequency error in magnetorheological finishing by simulation [15]. Wang et al. proposed a labyrinth path that increases randomness while ensuring a uniform distribution of trajectories and avoiding periodic surface errors [16]. Xu et al. studied the influence of tool spacing, polishing instability, and removal depth on the mid-spatial frequency error of magnetorheological finishing by computer simulation and a magnetorheological uniform scanning experiment [10]. Yan et al. used magnetorheological finishing to control the wave-front mid-spatial frequency error of large aperture planar optical components. The correction ability of the size of the magnetorheological finishing removal function to the spectral error and the suppression method of the magnetorheological finishing residual error was studied [17]. Jing et al. theoretically analyzed the main factors affecting MSF errors in grating path processing, and their study found that the removal depth and processing distance of single scanning were the main factors affecting the polishing effect under the condition of determining the polishing point [18]. Peng et al. combined magnetorheological finishing (MRF) with hydrodynamic effect polishing (HEP) to greatly suppress the surface spatial frequency error [19]. Wan et al. proposed a novel processing form of the magnetorheological finishing (MRF) tool and its generation mechanism. There are specific path angles and steps, with only tens of microns bandwidth (‘magic’ angle step), under which a surface without significant path ripple can be stably realized without affecting the convergence of other frequency errors [20]. However, previous studies mainly focus on optimizing the magnetorheological processing path to suppress the growth of mid-spatial frequency error. The influence of tool spacing, removal function size, and removal depth on the mid-spatial frequency error of the machined surface is studied. As shown in Figure 1, the effect of the stability of the removal function on the intermediate frequency error of the processed components due to the fluctuation of the ribbon during magnetorheological finishing is rarely studied. Therefore, this paper studies the causes of magnetorheological ribbon fluctuation and the influence of process parameters on it, and the influence of magnetorheological ribbon fluctuation on mid-spatial frequency error of machined surface is explored. These works will guide us to keep the ribbon fluctuation within a certain range by optimizing the process parameters so as to reduce the mid-spatial frequency band error caused by magnetorheological finishing of high-power laser components.

## 2. Experimental Equipment

**Figure 2:**
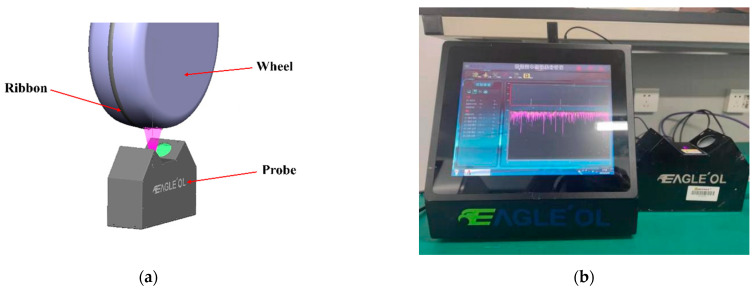
Magnetorheological polishing ribbon analysis device. (**a**) The schematic diagram of the magnetorheological polishing ribbon analysis device. (**b**) The magnetorheological polishing ribbon analysis device.

In magnetorheological finishing, the rheological properties, morphology, and stability of the magnetorheological fluid on the surface of the polishing wheel are important factors affecting the polishing performance. Therefore, it is necessary to monitor the stability of the polished ribbon in the polishing process to provide support for the study of stable polishing. Figure 2a shows the schematic diagram of the magnetorheological polishing ribbon analysis device. Figure 2b shows the magnetorheological polishing ribbon analysis device. The device uses machine vision to measure the cross-sectional profile of the polishing ribbon on the polishing wheel when the polishing wheel rotates. The device can measure the cross-sectional profile of the ribbon at the polishing position on the polishing wheel, including the height and width of the polished ribbon; the amplitude and frequency characteristics of fluctuations in the cross-sectional profile of the ribbon at any point. Its ribbon profile measurement accuracy is higher than 5 μm; repeatability accuracy is higher than 5 μm; measurement frequency is better than 600 Hz; measurement range is 2 mm along the running direction of the ribbon. Therefore, it can meet the measurement requirements of ribbons.

**Figure 3:**
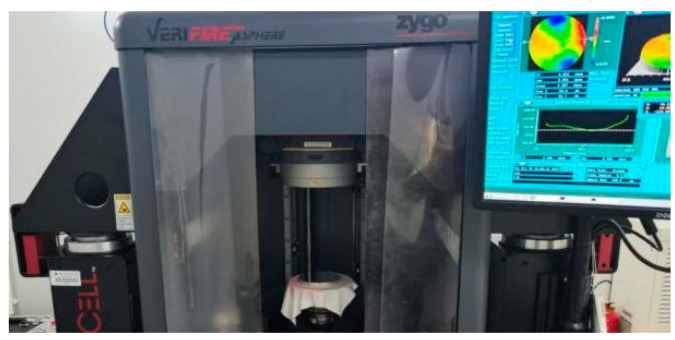
Six-inch aspheric vertical interferometer of Zygo VerFire Asphere.

In the mid-spatial frequency band error analysis of the machined optical component surface, a six-inch aspheric vertical interferometer of Zygo VerFire Asphere is used to measure the surface shape of the component, as shown in Figure 3. RMS of plane wave-front repeatability is less than 2 nm, and RMS of measurement repeatability is less than 0.05 nm.

## 3. Computer Simulation of the Influence of the Ribbon Fluctuation on the Mid-Spatial Frequency Error

### 3.1. Simulation Theory

An absolute plane was simulated and processed by Matlab. In the experiment, the plane was removed by uniform scanning. The removal function of magnetorheological finishing was obtained through the experiment, and the removal function was obtained when the average immersion depth of the ribbon was 0.2 mm. As the ribbon fluctuations change periodically, assuming the shape of the bottom surface of the removal function remains constant, the fluctuations in the ribbon will cause the depth of the ribbon immersion into the processed component to change, thus affecting the material removal efficiency and thus changing the removal function matrix. Let the ribbon periodic fluctuations be sinusoidal variations whose time-varying removal function matrix *R*1 is shown in Equation (1).

(1) R1=R×(1+|A×sin(f×pi×t×i|)

In the formula: *R* is the known two-dimensional removal function matrix; *A* is the amplitude of variation of removal efficiency caused by fluctuation in simulation; *f* is the period of removal function change; *t* is the simulated dwell time of each node; *i* is the number of points moved by the removal function.

The simulation material removal algorithm is the pulse-iterative method. The material in the polishing area of the removal function is concentrated to a point so that the removal function is in the form of a pulse. Furthermore, because the experiment adopts uniform scanning, each point’s dwell time is a fixed value, *t*(*x*, *y*). The residual error after material removal can be expressed as Equation (2).

(2) E(x,y)=M(x,y)−R1(x,y)**t(x,y)

### 3.2. Effect of Ribbon Fluctuation on Removal Efficiency

**Figure 4:**
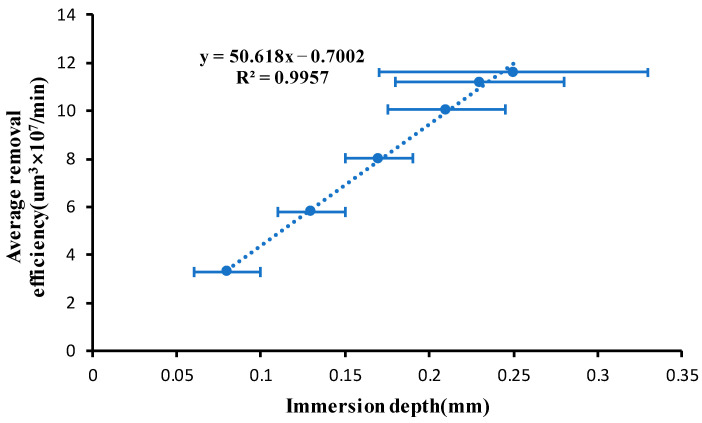
Relationship between average immersion depth and volumetric removal efficiency.

In order to explore the influence of ribbon fluctuation on the removal efficiency of magnetorheological finishing, we ensure that the distance between the polishing wheel and the surface of the component to be processed remains unchanged, and the immersion depth is changed by changing the flow rate. The speed of the polishing wheel is set to 220 rpm, and the flow rate increases from 120 Lph to 170 Lph. When the flow rate is 120 Lph, the immersion depth of the magnetorheological polishing ribbon is 0.1 mm, and the distance between the polishing wheel and the surface of the processed element remains unchanged with the change in flow rate. Because the ribbon is always fluctuating, we take the average value of instantaneous maximum immersion depth and minimum immersion depth to represent the material removal efficiency corresponding to the immersion depth. The relationship between the average immersion depth and the volume removal efficiency is shown in Figure 4.

From the above figure, the relationship between the average immersion depth and the volumetric removal efficiency is approximately linear. Therefore, when the ribbon fluctuates, the average removal efficiency corresponding to the instantaneous maximum immersion depth and the minimum immersion depth can be used to replace the average removal efficiency of the ribbon under this fluctuation.

### 3.3. Simulation Results

**Figure 5:**
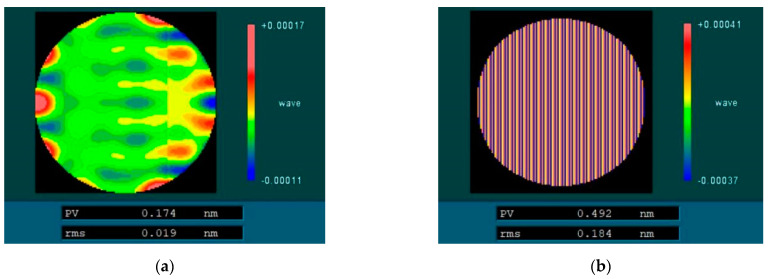
Surface mid-spatial frequency error after simulation processing. (**a**) Surface PSD1 error after simulation processing. (**b**) Surface PSD2 error after simulation processing.

**Figure 6:**
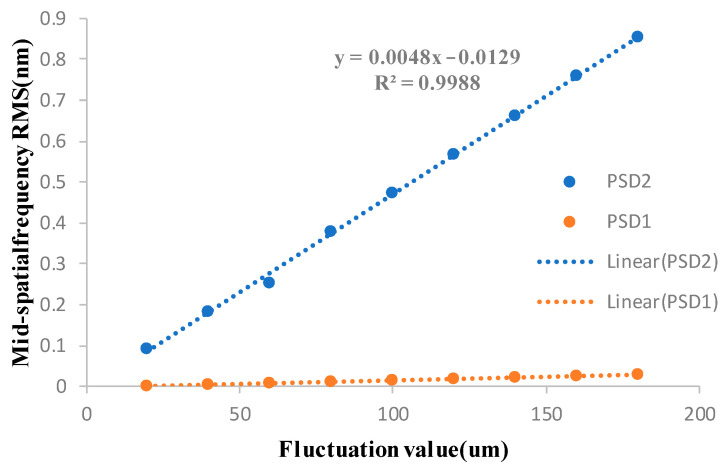
Effects of different ribbon fluctuations on PSD1 and PSD2 in the mid-spatial frequency band.

The removal function with an average immersion depth of 0.2 mm is selected to process an absolute plane, and the removal depth is 0.5 μm. The influence of different ribbon fluctuations on the plane mid-spatial frequency error is analyzed by computer simulation. Figure 5 shows that the surface intermediate frequency error is simulated when the ribbon fluctuation is 40 μm. Figure 6 shows the influence of different ribbon fluctuations on PSD1 and PSD2 in the mid-spatial frequency band. The line spacing of the magnetorheological uniform scan is 1 mm, and the spatial frequency of 1 mm^−1^ is included in the PSD2 band, so the RMS of the PSD2 range will be affected by the mid-spatial frequency error generated by the line spacing, so the mid-spatial frequency band of PSD2 analyzed in this paper is 0.4–0.9 mm^−1^.

From the above figure, it can be analyzed that the ribbon fluctuations caused by the fluctuation of the removal amount on the surface of the processed components in the mid-spatial frequency band PSD1 RMS have a small impact; the impact on the mid-spatial frequency band PSD2 RMS is significant, with the ribbon fluctuations increasing in line with the increasing mid-spatial frequency band PSD2 RMS, its growth is basically linear, so to control the error of the surface in the middle frequency band PSD2 need to control the ribbon fluctuations.

## 4. The Causes of Ribbon Fluctuation and the Influence of Process Parameters on Ribbon Fluctuation

### 4.1. The Causes of Ribbon Fluctuation

The magnetorheological finishing process requires that the magnetorheological fluid ejected from the nozzle is apparent on the surface of the polishing wheel, and a polishing ribbon for polishing is formed under the action of the magnetic field around the polishing wheel. Therefore, the reason for ribbon fluctuation may be the change in the magnetic field on the surface of the polishing wheel or the fluctuation of the magnetorheological fluid sprayed from the nozzle.

**Figure 7:**
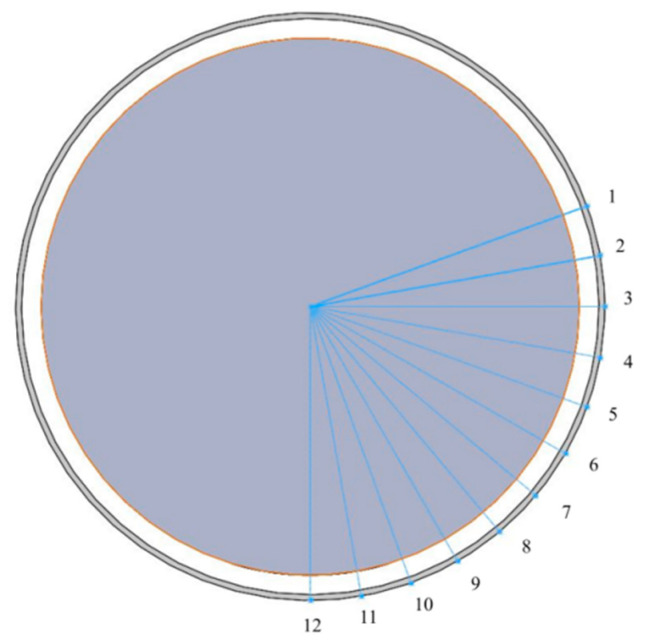
Magnetic field test path along circumference direction.

When exploring the influence of the magnetic field on the ribbon fluctuation of the polishing wheel surface, a Tesla meter is used to test the magnetic field strength of the magnetorheological polishing wheel surface. The measurement method is shown in Figure 7. The measurement is carried out along the circumference of the surface of the polishing wheel. The starting point of the measurement is the incident point of the magnetorheological polishing liquid entering the polishing wheel, and then it is measured every 10° until the lowest point of the polishing wheel.

**Figure 8:**
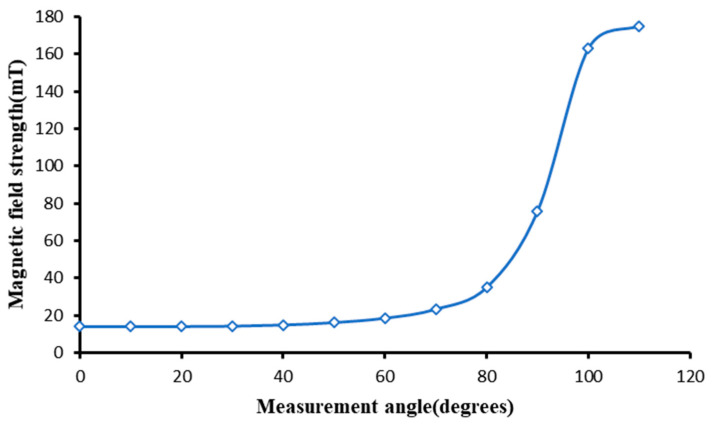
Distribution of circumferential magnetic field intensity.

The purpose of the existence of the magnetic field along the circular surface of the polishing wheel is to ensure that the magnetorheological fluid can be stably adsorbed on the surface of the polishing wheel before reaching the polishing area. After leaving the polishing area, the magnetorheological fluid is smoothly taken to the recycler and recycled into the recycling system to be used again. Figure 8 shows the surface magnetic field distribution along the circular direction.

The figure shows that the magnetic field strength of the magnetorheological fluid just entering the surface of the polishing wheel is about 18 mT, which meets the requirement of stable adsorption of the magnetorheological fluid on the surface of the polishing wheel, and the magnetic field strength increases steadily and slightly before reaching the polishing area along the circumference of the wheel and reaches 170 mT at the polishing area to meet the polishing requirements [21]. Moreover, the magnetic field strength at each position was found to remain constant without fluctuations in the test.

**Figure 9:**
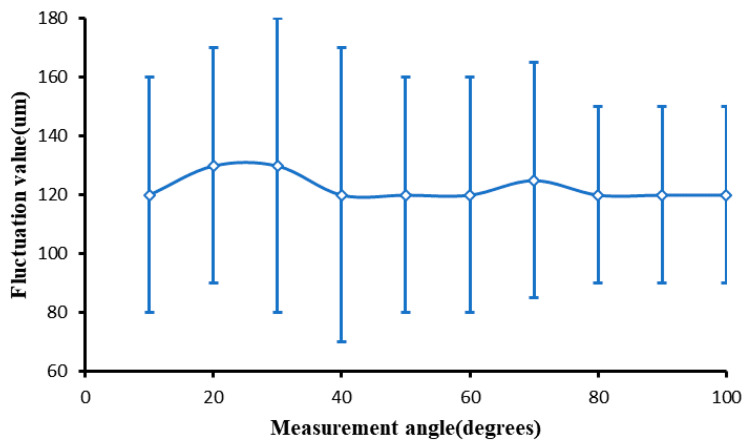
Variation of ribbon fluctuation along the surface of the polishing wheel.

In order to further study the influencing factors of magnetorheological ribbon fluctuation, we set the polishing wheel speed as 180 rpm and the flow rate as 130 Lph to measure the ribbon fluctuation at 12 positions along the polishing wheel surface, as shown in Figure 7. The experimental results of the ribbon fluctuation are shown in Figure 9.

It can be found that the ribbon fluctuation is stable along the circumferential surface of the magnetorheological polishing wheel until the polishing area. Combined with the analysis of the stability of the magnetic field intensity, it is proved that the cause of the ribbon fluctuation is independent of the magnetic field.

Subsequently, we conducted a profile test on the liquid column that the magnetorheological fluid was sprayed from but which did not reach the polishing wheel. It was found that the fluctuation of the liquid column was about 60 μm, and when the magnetorheological fluid was incident on the surface of the polishing wheel, the ribbon shape was no longer cylindrical. We assumed it to be a rectangular shape, and according to the rule of constant volume, Formula 3 can be obtained:

(3) L=k×a×b×v

In the formula: *L* is the flow rate of magnetorheological fluid; *k* is the volume correction coefficient (since the ribbon cross-section is not rectangular, *a* correction factor is needed); *a* is the width of the ribbon; *b* is the height of the ribbon; *v* is the rotating speed of the polishing wheel.

Due to the fluctuation of the liquid column, the volume difference caused by the fluctuation will be the main factor affecting the fluctuation of the magnetorheological liquid ribbon. Therefore, according to the constant volume difference, Formula 4:

(4) ΔV=k×Δa×b×v

In the formula: Δ*V* is the volume change caused by the fluctuation of the magnetorheological fluid column; Δ*a* is the fluctuation value of the ribbon.

**Figure 10:**
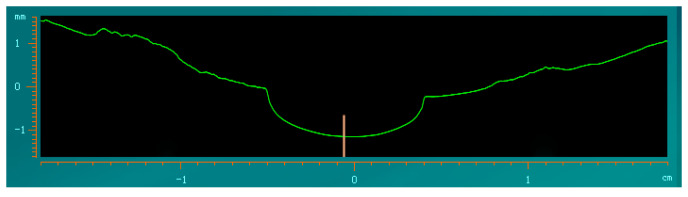
Morphology of magnetorheological polishing ribbon.

**Figure 11:**
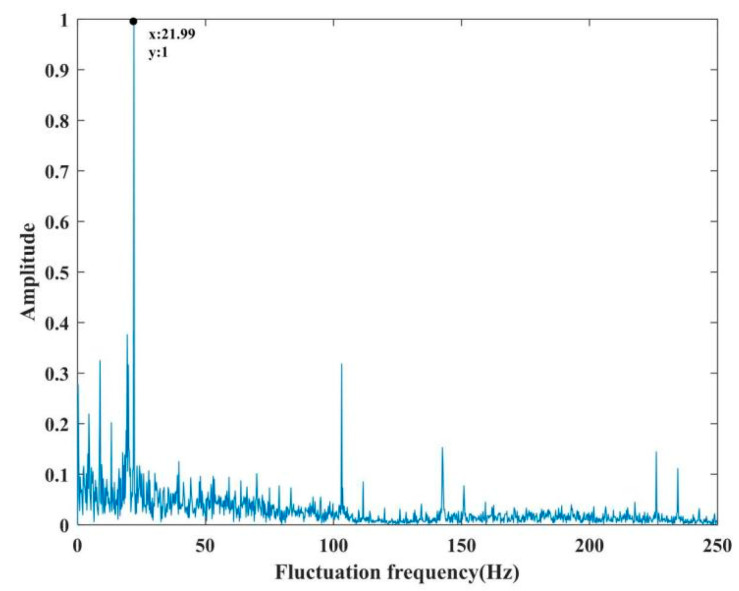
Fluctuation frequency of magnetorheological polishing ribbon.

Finally, the fluctuation frequency of the magnetorheological polishing ribbon in the polishing area is tested. When the polishing wheel speed is 260 rpm and the flow rate is 120 Lph, the instantaneous value of ribbon fluctuation is 70 μm. Figure 10 shows the ribbon morphology measured by the magnetorheological ribbon analyzer. Figure 11 shows the frequency distribution of the ribbon fluctuation at the orange line segment shown in Figure 10. The corresponding main frequency is 21.99 Hz, and the centrifugal pump frequency is also 21.99 Hz. Through many tests, it was found that the main frequency of ribbon fluctuation was the frequency of the centrifugal pump. It can be analyzed that the main factor affecting ribbon fluctuation is the flow fluctuation caused by the centrifugal pump when pumping liquid.

### 4.2. The Causes of Ribbon Fluctuation

#### 4.2.1. The Effect of Process Parameters on Ribbon Fluctuation

**Figure 12:**
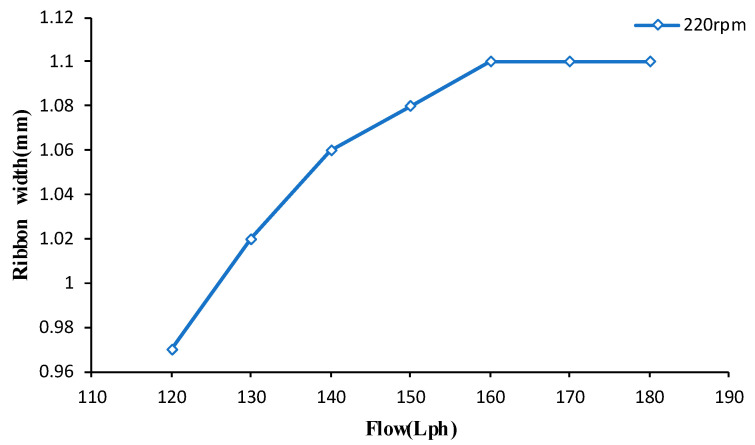
The variation of ribbon width with flow rate.

Firstly, the change in the ribbon size with flow rate is measured. Since it is difficult to measure the change in ribbon fluctuation thickness at any time, only the change of ribbon width with flow rate was measured, as shown in Figure 12.

It can be seen from Figure 12 that the ribbon width increased with the increase in the flow rate, but when the flow rate reached a certain value, the width did not change due to the shape of the magnetic field.

**Figure 13:**
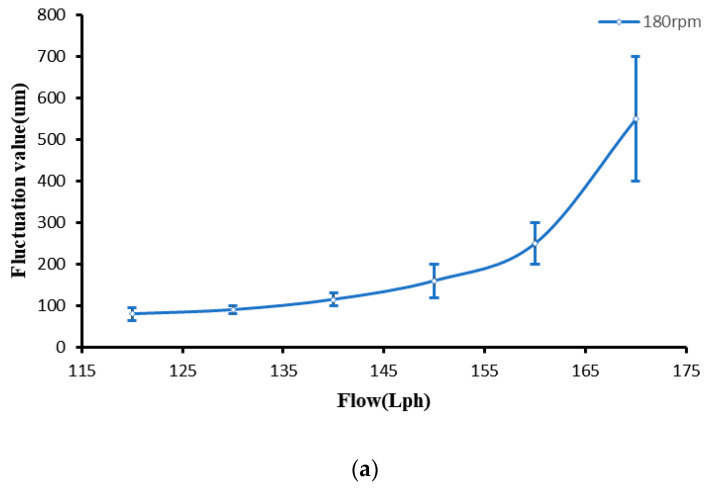

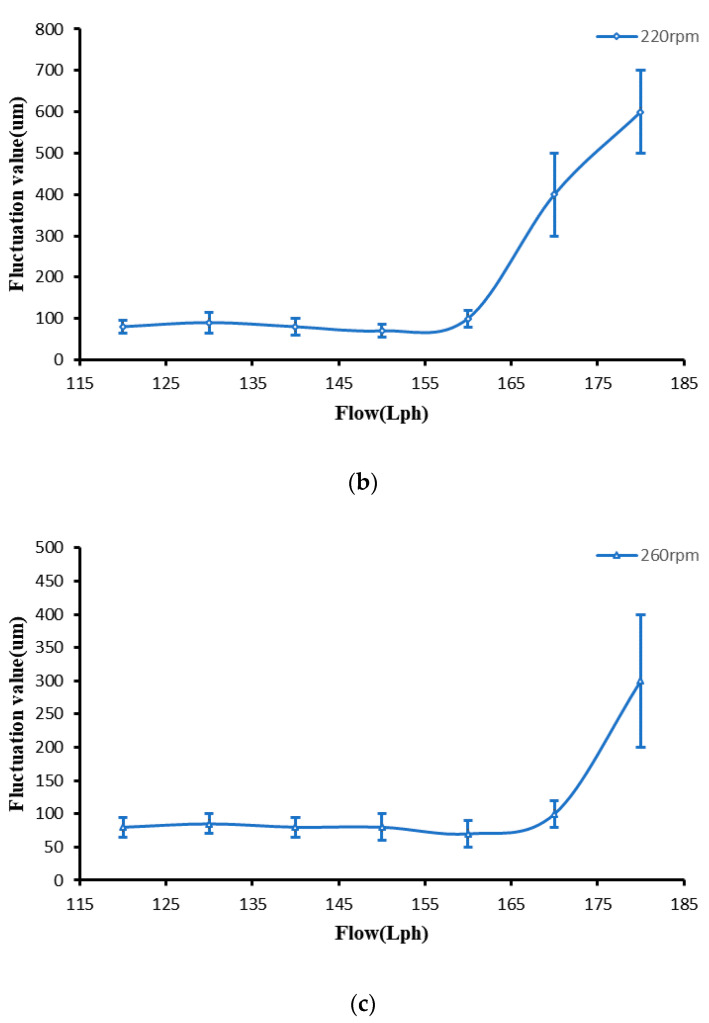
Variation of ribbon fluctuation with magnetorheological fluid flow. (**a**) Rotating speed 180 rpm. (**b**) Rotating speed 220 rpm. (**c**) Rotating speed 260 rpm.

Then we tested the variation in ribbon fluctuation with the flow rate. When we measured the effect of magnetorheological fluid flow rate on ribbon fluctuation, we kept the polishing wheel speed constant, changed the magnetorheological fluid flow rate at 180 rpm, 220 rpm, and 260 rpm, and tested the relationship between ribbon fluctuation and flow rate; the ribbon fluctuation change is shown in Figure 13.

It can be seen from the above figure that when the flow rate remains small, the ribbon fluctuation remains in a low range, but as the flow rate continues to increase, the ribbon fluctuation will increase significantly. By testing the magnetorheological profile of the unreached polishing wheel, it was found that the fluctuation in the liquid column increased gradually with the change in flow rate. Flow fluctuations became larger, but when the flow remained small, with the increased flow, ribbon width increased, as shown in Figure 12. According to Formula 4, due to the increase in width and flow fluctuation, the fluctuation of the ribbon is basically constant. However, when the flow increases to a certain value, the width of the ribbon is no longer increased due to the distribution of the magnetic field. When the flow fluctuation becomes larger, the fluctuation of the ribbon increases significantly.

#### 4.2.2. The Effect of Rotating Speed of Polishing Wheel on Ribbon Fluctuation

**Figure 14:**
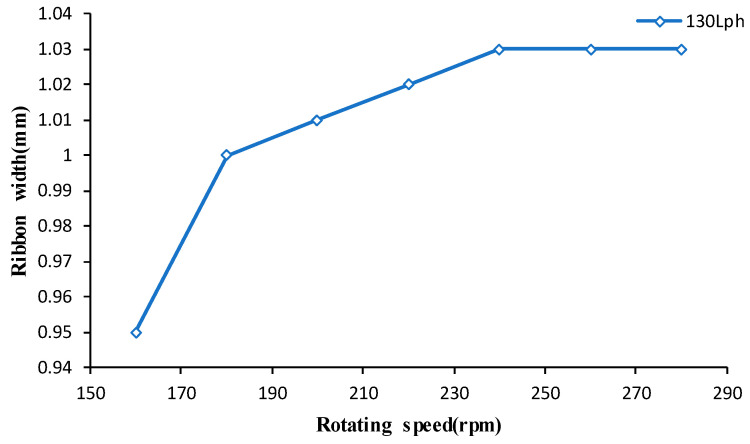
The variation of ribbon width with rotating speed.

Firstly, the variation of ribbon width with the rotating speed of the polishing wheel is measured, as shown in Figure 14.

It can be seen from Figure 14 that the ribbon width increases with the increase in rotating speed, but when the rotating speed reaches a certain value, the width does not change due to the shape of the magnetic field.

**Figure 15:**
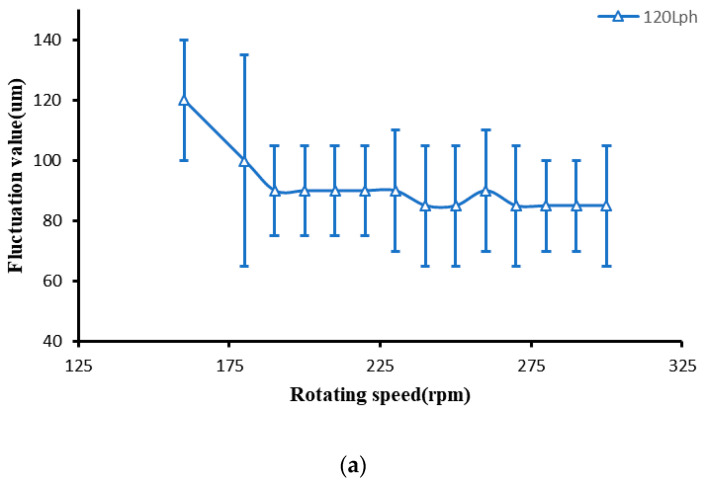

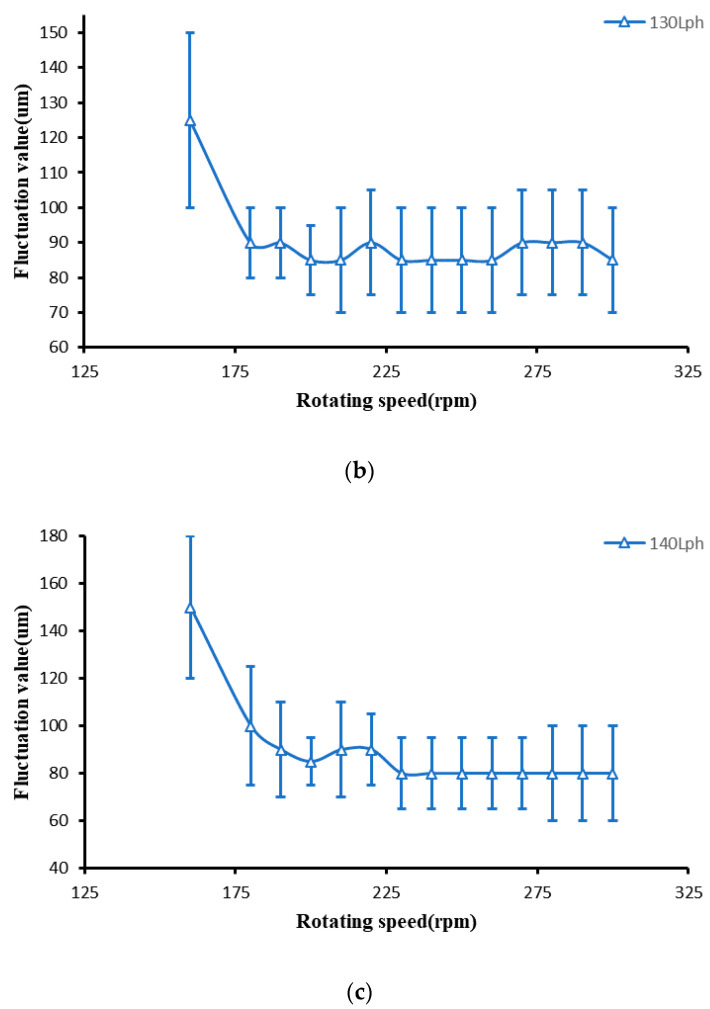
Variation in ribbon fluctuation with the rotating speed of the polishing wheel. (**a**) Flow rate 120 Lph. (**b**) Flow rate 130 Lph. (**c**) Flow rate 140 Lph.

In the study of the influence of the rotating speed of the polishing wheel on ribbon fluctuation, we kept the flow rate of the magnetorheological fluid unchanged and only changed the rotating speed of the polishing wheel. The relationship between the ribbon fluctuation and the rotating speed of the polishing wheel was measured when the flow rates of magnetorheological fluid were 120 Lph, 130 Lph, and 140 Lph. The variation in ribbon fluctuation is shown in Figure 15.

It can be seen from the above figure that when the rotating speed of the polishing wheel is low, the ribbon fluctuates greatly. Furthermore, when the rotating speed of the polishing wheel increases again, the ribbon fluctuation basically remains stable. Since the flow rate remains unchanged, the flow fluctuation remains unchanged. When the rotating speed is low, the width of the magnetorheological ribbon is small, as shown in Figure 14. At the same flow fluctuation, according to Formula 4, the fluctuation of the ribbon is large. When the speed of the polishing wheel continues to increase, the width of the band increases to the limit width and does not increase anymore due to the magnetic field distribution. The flow fluctuation remains stable, so the fluctuation of the band also remains stable.

## 5. The Effect of Ribbon Fluctuation on Mid-Spatial Frequency Error in Magnetorheological Finishing

### 5.1. Experiments and Results

**Figure 16:**
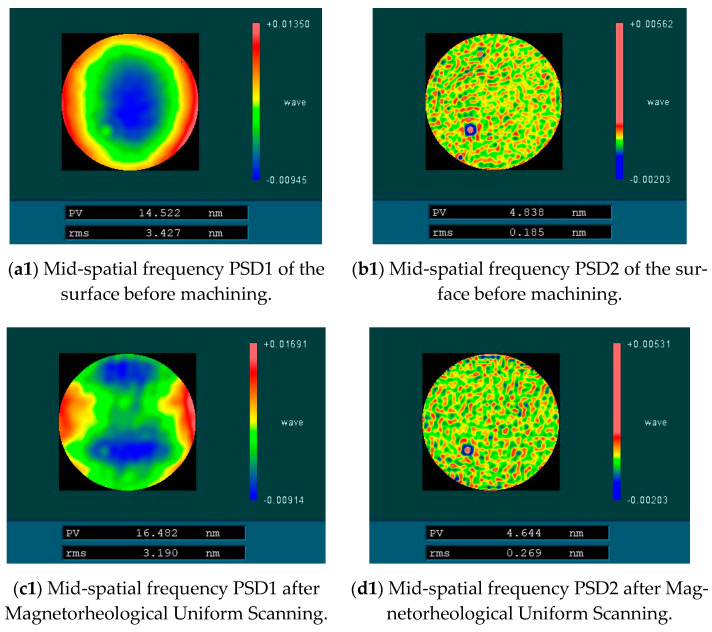

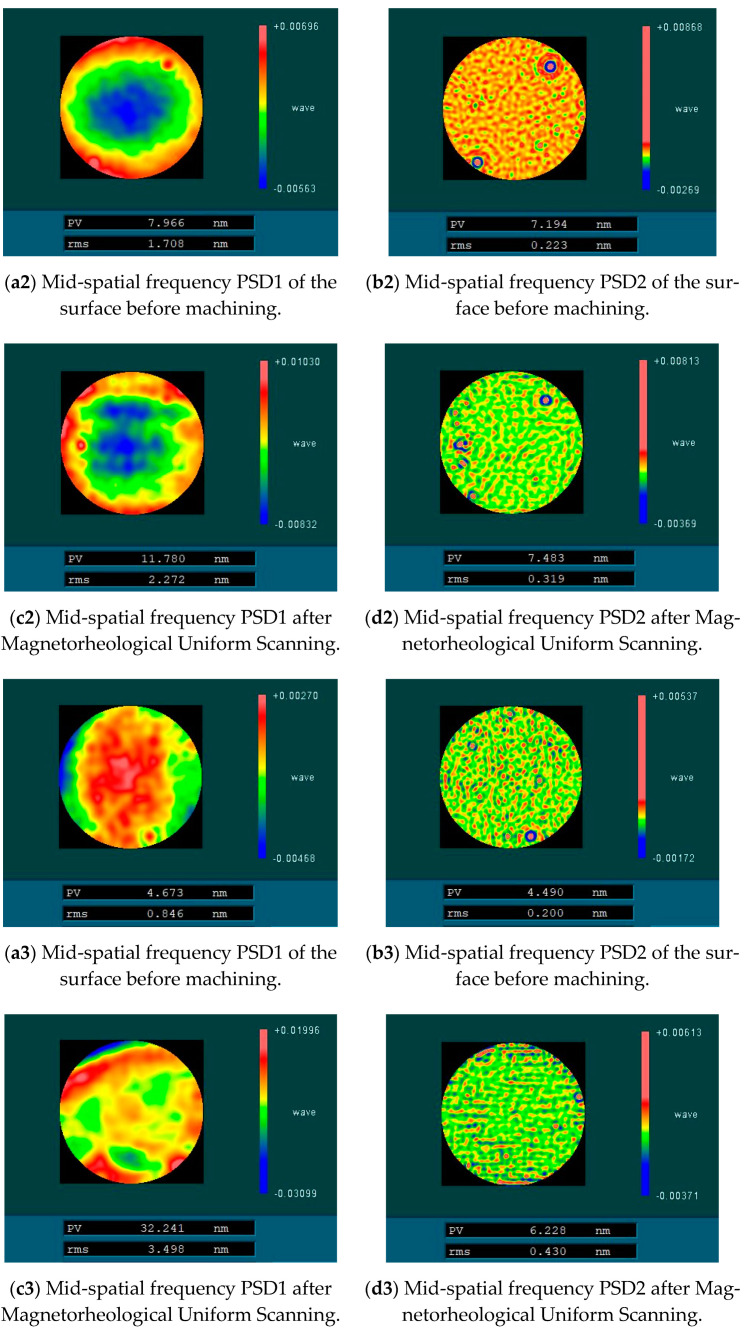

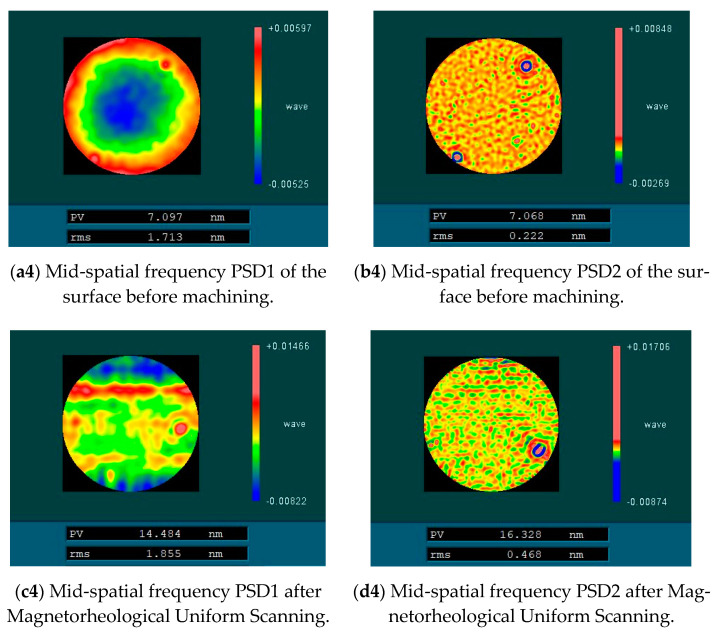
Mid-spatial frequency error before and after magnetorheological processing.

**Figure 17:**
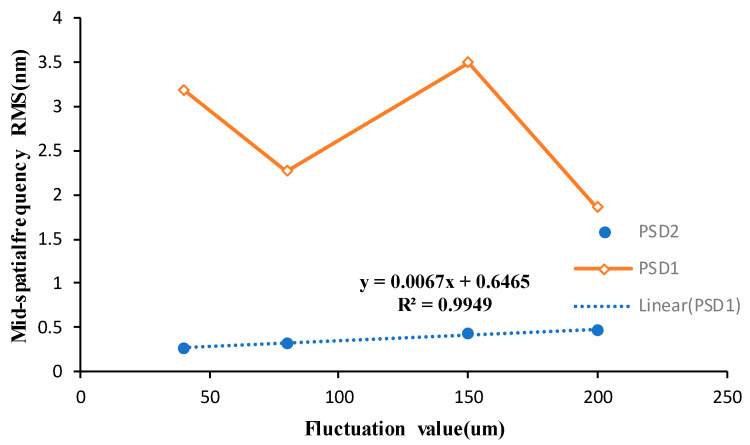
Effects of different ribbon fluctuations on mid-spatial frequency PSD1 and PSD2.

The uniform scanning experiment of four fused silica elements with similar surface mid-spatial frequency quality was carried out by magnetorheological finishing. During the experiment, the distance between the polishing wheel and the processing element was kept constant so that the magnetic field intensity at the surface of the element was kept constant, and the material removal depth was kept constant during the four processes. The four experimental parameters are shown in Table 1. After processing, the surface RMS of the components in the mid-spatial frequency bands PSD1 0.0303–0.4 mm^−1^ and PSD2 0.4–0.9 mm^−1^ were tested. Figure 16 shows RMS values of PSD1 and PSD2 on the surface of four fused silica components before and after magnetorheological processing. Figure 17 shows the variation of PSD1 and PSD2 with ribbon fluctuation after actual processing.

The experimental results show that with the increase in ribbon fluctuation, the RMS value of PSD2 in the mid-spatial frequency band on the machined component surface increases continuously, and due to the influence of the initial surface shape, the RMS value changes approximately linearly, which is basically consistent with the simulation results. For the RMS value of PSD1 in the mid-spatial frequency band on the component surface, the shape and size of the removal function are changed due to the change of flow rate and the fluctuation of ribbon in the experiment, and the smaller removal function has a good correction ability [17], so the size of the removal function is constantly changing, which is different from the simulation results.

**Figure 18:**
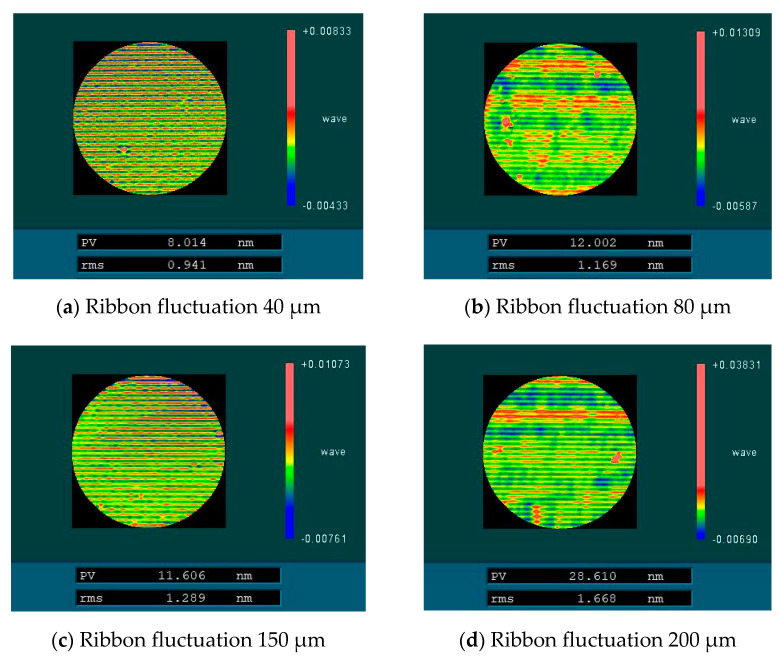
Effects of different ribbon fluctuations on the full frequency RMS of PSD2.

Finally, the influence of different ribbon fluctuations on the full-frequency RMS of PSD2 on the machined fused silica element surface is tested, as shown in Figure 18. The experimental results show that in the actual processing, the step distance produced by magnetorheological finishing had an impact on the mid-spatial frequency PSD2. It is necessary to control the process parameters and control the ribbon fluctuation to remain below 80 μm to meet the RMS of PSD2 less than 1.1 nm.

### 5.2. Discussion

From the results above, it can be seen that the fluctuation of the magnetorheological polishing ribbon during the magnetorheological polishing process will produce mid-spatial frequency errors on the surface of the component. Moreover, the main reason for ribbon fluctuation is the change in magnetorheological fluid flow caused by centrifugal pump rotation in the magnetorheological circulation system. When the flow rate of the magnetorheological fluid is increased by controlling the speed of the centrifugal pump, the ribbon fluctuation will increase. However, if a lower flow rate is used, the width and thickness of the magnetorheological fluid ribbon are smaller, which leads to a smaller removal function shape, seriously affecting the removal efficiency of the material. Furthermore, the magnetorheological fluid flow rate cannot be too high due to the upper limit of the centrifugal pump speed. As for the polishing wheel speed, the ribbon fluctuation decreases with the increase in the polishing wheel speed. The width of the ribbon increases as the speed increases, but the width does not change when the speed reaches a certain value due to the shape of the magnetic field. At this time, when the polishing wheel speed increases, ribbon fluctuations remain basically constant. When the polishing wheel speed increases, the thickness of the ribbon decreases, so the polishing wheel speed can not be too large. Because the mid-spatial frequency error on the surface of the component increases with the increase in ribbon fluctuation, it is necessary to control the flow of magnetorheological fluid and the speed of the polishing wheel to control the fluctuation of the ribbon in order to meet our requirements for the surface mid-spatial frequency error and the width and thickness of the ribbon during the actual processing.

## 6. Conclusions

In this paper, the causes of ribbon fluctuation in magnetorheological finishing and its influence on the surface mid-spatial frequency error of machined components are studied. The correlation between ribbon fluctuation and magnetic field and the centrifugal pump is analyzed. It is proved that the causes of ribbon fluctuation are independent of the magnetic field. The main reason is the change in magnetorheological fluid flow caused by the centrifugal pump rotation in the magnetorheological circulation system. The influence of magnetorheological fluid flow rate and polishing wheel speed on ribbon fluctuation was explored. When the polishing wheel speed is constant and the magnetorheological fluid flow rate is small, the ribbon fluctuation basically remains unchanged with the increase in flow rate, but when it increases to a certain value, the ribbon fluctuation increases with the increase in flow rate. When the flow rate of the magnetorheological fluid is constant, the ribbon fluctuation decreases significantly with the increase in the rotating speed of the polishing wheel. However, when the rotating speed of the polishing wheel increases to a certain value, the ribbon fluctuation remains unchanged with the increase in the rotating speed of the polishing wheel. Finally, the influence of ribbon fluctuation on the surface mid-spatial frequency error of the machined component is studied by computer simulation and actual machining. The experimental results show that with the increase in ribbon fluctuation, the RMS value of the mid-spatial frequency PSD2 on the machined component surface increases continuously and changes approximately linearly due to the influence of the initial surface shape. For the RMS value of PSD1 in the mid-spatial frequency band on the component surface, the shape and size of the removal function are changed due to the change in the flow rate and the fluctuation of the ribbon in the experiment, and the size of the removal function is constantly changing, which makes it different from the simulation results. In summary, the fluctuation of the polishing ribbon can be controlled by optimizing the flow rate of the magnetorheological fluid and the rotating speed of the polishing wheel so as to control the quality of the mid-frequency band of the machined surface.

## Figures and Tables

**Table 1:** Experimental parameters.

Item	Experiment 1	Experiment 2	Experiment 3	Experiment 4
Rotating speed	220 rpm	220 rpm	220 rpm	220 rpm
Flow	140 Lph	160 Lph	170 Lph	180 Lph
Ribbon fluctuation	40 μm	80 μm	150 μm	200 μm

## Data Availability

The data presented in this study are available on request from the corresponding author. The data are not publicly available due to the data also forms part of an ongoing study.
